# Sole-Leather Splints—an Improved Process for Making Them

**Published:** 1880-10

**Authors:** Bernard Bartow


### SOLE-LEATHER SPLINTS.—AN IMPROVED PROCESS FOR MAKING THEM.

BY BERNARD BARTOW, M. D.

THE use of sole-leather for splints and other surgical appliances, though commonly mentioned by surgical writers, has never reached great popularity owing to the difficulty attending its ready manipulation. Possessing as it does, strength, lightness, durability and porosity—almost every requisite for a perfect splint—it is unfortunate that it should be lacking in pliability,—which constitutes the principal objection to its more general use. This difficulty, however, of moulding leather upon complicated forms, is only a comparative one, for when the necessary force can be employed, it can be made to assume quite easily, almost any desired shape.

As ordinarily used, a splint has been formed by moulding a previously moistened piece of leather, upon the limb which it was designed to support. In such cases, the limb being injured, or in some other abnormal state, only a slight degree of force could be used in forming it; the result would be an imperfectly fitting, clumsy support. Even though the limb were normal and able to bear considerable pressure, the yielding nature of the muscular tissue would prevent the formation of, what is a desideratum in a splint, viz., the counterpart of the limb in form.

These objections to the free use of leather for splints, I have in large part overcome, by substituting for the human limb, accurate casts in plaster of Paris, of human limbs. These furnish resisting forms upon which the leather, previously moistened and rendered flexible, can be moulded by the application of the requisite force, so that a splint made in the manner I shall describe shall be a counterpart of the form upon which it is made. To make a cast of a limb—taking for example the leg and foot, I first make a mould in the following manner: The limb is freely anointed with olive oil, after which a tight fitting stocking may be drawn upon it (a bandage nicely applied from the toes to the knee answers nearly as well as the stocking.) For an adult limb, take one pound of bee's wax and melt it by placing it in a basin of boiling water, when the melted wax will rise to the surface. With a flat paint brush, evenly and quickly apply the melted wax to the limb, the limb being held in the proper position by an assistant, taking care to apply it thinly when putting on the first layer, to avoid burning the model. After the limb is thinly covered with wax it may be applied quite freely without burning. When a coating one-eighth of an inch thick has been formed upon the limb, cut through the stocking and wax upon the front of the leg, from the knee to the toe, and carefully spring the mould off the limb. Adjust the cut edges and fasten with a few strips of cloth dipped in hot wax, after which the mould is evenly bandaged to give it additional strength, and then placed in a box or keg containing light loam or sand which is placed around the mould, to support it evenly while the plaster is being poured in. After the plaster has "set" the mould may be removed from the casting in the same manner as from the limb, and laid aside for subsequent use. For the splint, a piece of sole-leather from one-sixteenth to one-fourth of an inch in thickness (according to the strength necessary for the splint) sufficient to invest the casting, is required. Before applying this, it should be soaked from one to three hours in warm water until it is quite soft and flexible, then wrap it around the casting, cutting away the leather until the edges meet accurately in front, at the top of the cast, at which point it should be fastened with a strong cord. Then with a long piece of marline or clothes-line closely wind the leather upon the cast, cutting away, in advance of the cord, the superfluous leather as it is drawn to the front, making the edges meet in the centre of the cast in front; in this manner continue over the entire surface of the leather, which will be drawn in, and made to conform to every part of the surface beneath it. Place the whole in an oven to dry. Then remove the leather case from the cast, and cut it down the centre behind, making two side splints. After cutting away the sharp edges, the splint is ready for use.

A number of castings of limbs, from persons of different ages and sizes, may, in this manner be made and upon them may be formed splints, which would be adapted to all ordinary uses. Their durability enables them to be used an indefinite number of times, and even though they may be moistened frequently, in fitting them to different individuals, they retain their form. With them, the ease with which a fractured limb may be dressed, is reduced to a minimum, as is also the subsequent care that a case of fracture requires. The splint being the exact mould of a a limb in proper position, a fractured limb once encased with it, is firmly and accurately held in the proper position for the bones to unite. Extension is constantly maintained by reason of the splint taking hold of the whole surface of the limb. Either the scultetus or many-tailed bandage may be employed to secure the splint, which will allow of easy examination of the limb with the least possible disturbance. The patient may be permitted to go upon crutches in four weeks with such a dressing, the lightness of which is no inconsiderable feature in the comfort he enjoys in this connection. In one case of fracture of both bones of leg occurring in the practice of a friend, the patient was permitted to go about on crutches at the end of fourteen days with this form of dressing. It is not claiming too much for it, to make the statement, that it will wholly take the place of plaster of Paris as a permanent dressing for fractures.

I can recall twenty-five cases of fracture of the leg in which I have employed this form of splint, or seen it employed; and in every instance it has given eminent satisfaction to both patient and physician. I know of no case where deformity has followed its use, and in no case where it has been used has the shortening of a limb exceeded one-half inch, while in many cases no shortening was discoverable. A number of surgeons of this city have employed it, and all report the most gratifying results from its use.

The same process I have described for making splints, would, it seems to me, be applicable for making the corset or jacket for spinal curvature. This appliance made of leather would possess many advantages over plaster of Paris, or the "paper and glue" brace of Dr. Morgan Vance. It could be easily removed for purposes of cleanliness, as the degree of curvature changed it could be remoulded to accord with the changes, while its expensiveness to begin with, might exceed that of other methods, its durability, and the ability to use it over and over, would in the end make it the most economical apparatus.

